# Evolution of Genetic Potential

**DOI:** 10.1371/journal.pcbi.0010032

**Published:** 2005-08-26

**Authors:** Lauren Ancel Meyers, Fredric D Ancel, Michael Lachmann

**Affiliations:** 1 Section of Integrative Biology, Institute for Cellular and Molecular Biology, University of Texas, Austin, Texas, United States of America; 2 Santa Fe Institute, Santa Fe, New Mexico, United States of America; 3 Department of Mathematical Sciences, University of Wisconsin, Milwaukee, Wisconsin, United States of America; 4 Max Planck Institute for Evolutionary Anthropology, Leipzig, Germany; Pennsylvania State University, United States of America

## Abstract

Organisms employ a multitude of strategies to cope with the dynamical environments in which they live. Homeostasis and physiological plasticity buffer changes within the lifetime of an organism, while stochastic developmental programs and hypermutability track changes on longer timescales. An alternative long-term mechanism is “genetic potential”*—*a heightened sensitivity to the effects of mutation that facilitates rapid evolution to novel states. Using a transparent mathematical model, we illustrate the concept of genetic potential and show that as environmental variability decreases, the evolving population reaches three distinct steady state conditions: (1) organismal flexibility, (2) genetic potential, and (3) genetic robustness. As a specific example of this concept we examine fluctuating selection for hydrophobicity in a single amino acid. We see the same three stages, suggesting that environmental fluctuations can produce allele distributions that are distinct not only from those found under constant conditions, but also from the transient allele distributions that arise under isolated selective sweeps.

## Synopsis

Variation is the fuel of natural selection. Understanding the mutational processes that underlie evolution has long been a central objective of population genetics. Today, amidst a computational revolution in biology, such understanding is pivotal to progress in many biological disciplines. For example, neutral mutations make the molecular clock tick, and this clock is fundamental to reconstructing phylogenies, measuring recombination rates, and detecting genetic functionality. In this manuscript, the researchers provide an original perspective on a long-standing question in evolutionary biology: to what extent do mutation rates evolve? They argue that to cope with environmental fluctuation, populations can evolve their phenotypic mutation rate without changing their genetic mutation rate. That is, populations can evolve “genetic potential”—a heightened sensitivity to the effects of mutation. The researchers use a simple mathematical model of amino acid evolution to illustrate the evolution of genetic potential, and show that as environmental variability decreases, evolving populations reach three distinct states. In a rapidly fluctuating environment, organisms evolve the flexibility to cope with variation within an individual lifetime; in moderately variable environments, populations evolve the ability to evolve rapidly; and in fairly constant environments, populations evolve robustness against the adverse effects of mutation.

## **Introduction**

Recent work in evolutionary biology has highlighted the degeneracy of the relationship between genes and traits [1]. For any particular trait value, there will exist a large set of genotypes that give rise to that value. A mutation from one such genotype to another will be neutral, having no noticeable impact on the physiology, behavior, or fitness of organisms. Metaphorically, one can imagine a population moving via mutation through a region of genotype space that maps to a neutral plateau in phenotype space. Near the periphery, mutations are likely to produce different (usually worse and occasionally better) phenotypes, whereas near the center of the neutral plateau, mutations have little impact on the phenotype. Evolutionary theory suggests that populations can harness this variation to achieve phenotypic stability under steady conditions through either mutational insensitivity [2,3] or mutational hypersensitivity [4], or to facilitate phenotypic exploration during adaptation [5,6].

A separate body of evolutionary theory addresses adaptation under fluctuating conditions [7,8]. The rate of the fluctuations will influence the resulting response. If the environment changes rapidly relative to the average generation time, populations may evolve mechanisms such as physiological plasticity and learning by which individual organisms can respond to their conditions [9,10]. As environmental change slows down, viable strategies include stochastic or directed heterogeneity in developmental pathways that give rise to phenotypic variation on the order of once per generation [11]. For even slower rates of change, mutations may produce novel phenotypes at a sufficiently high rate. Hypermutable lineages can produce novelty every few generations, as has been observed in viruses and mutator strains of bacteria [12,13]. When environmental fluctuations are rare, populations may experience extended epochs of directional selection and thus have sufficient time to achieve genetic robustness for any given state. Immediately following an environmental shift, however, such populations may pass through transitional periods of within-individual or between-generation plasticity before completely losing the previously favored phenotype in favor of a currently favored phenotype. This evolutionary transformation—from a trait that is acquired through phenotypic plasticity to a genetically determined version of the same trait—is known as the Baldwin Effect [9,14].

In this paper we show that genetic degeneracy may give rise to an alternative outcome under fluctuating conditions: the evolution of genotypes with heightened sensitivity to mutation. We introduce the term “genetic potential” to describe this state. Metaphorically, populations with genetic potential lie near the edge of neutral plateaus. Although the rate of mutation is unchanged, the likelihood that mutations produce beneficial variation increases. Heightened sensitivity to mutations has been recognized as a critical and transient phase of adaptive evolution [5,15,16]. Here we argue that genetic potential can be a stable condition for a population evolving under changing selection pressures. Using a simple mathematical model, we show that as environmental variability increases, natural selection at first moves populations between genetically robust states, then increasingly favors genetic potential, and ultimately produces mechanisms for environmental robustness within individual organisms.

We then present a more biological example of this phenomenon using a model of amino acid evolution. There is evidence that, within viral pathogens, the physiochemical properties of amino acids found within epitopes—regions of proteins that directly interact with the host immune system—can rapidly evolve [17,18]. Likewise, highly evolvable codons have been identified in bacteriophage experiencing shifting hosts [19] and in enzymes experiencing shifting substrates [20]. Motivated by these observations, we model codon evolution at a single amino acid site under fluctuating selection for hydrophobicity. As in the first model, natural selection produces three distinct outcomes with increasing environmental variability. Each outcome corresponds to distinct expectations about the distribution of amino acids and their codons at selected sites.

**Figure 1:**
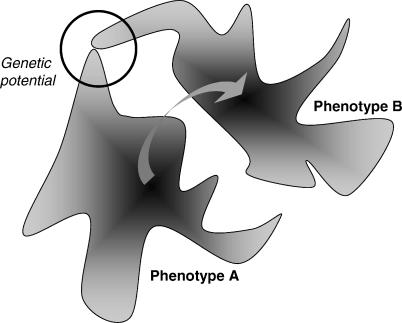
Evolution of Genetic Potential The gray regions represent neutral networks—sets of genotypes that give rise to each phenotype. The degree of shading indicates the likelihood that mutations will impact phenotype, where darker regions are robust to mutations. Under constant conditions, populations evolve toward the most robust regions of neutral networks. Under variable conditions, populations may evolve toward genotypes that easily mutate from one phenotype to the other. These regions of genetic potential do not always lie on the evolutionary path between the equilibrium states for constant environments (arrow).

Under infrequent environmental change, populations evolve from one mutationally robust phenotype to another, briefly passing through genotypes that can easily mutate to either state. One might therefore be tempted to equate genetic potential with confinement to the intermediate steps on a path from robustness for one phenotype to robustness for another (Figure 1). While this is true in our simple model, the codon model illustrates that fluctuating environments may drive populations towards significantly greater genetic potential than found during these transient stages of isolated selective sweeps.

## Results

### Description of Models

#### The simple model.

**Figure 2:**
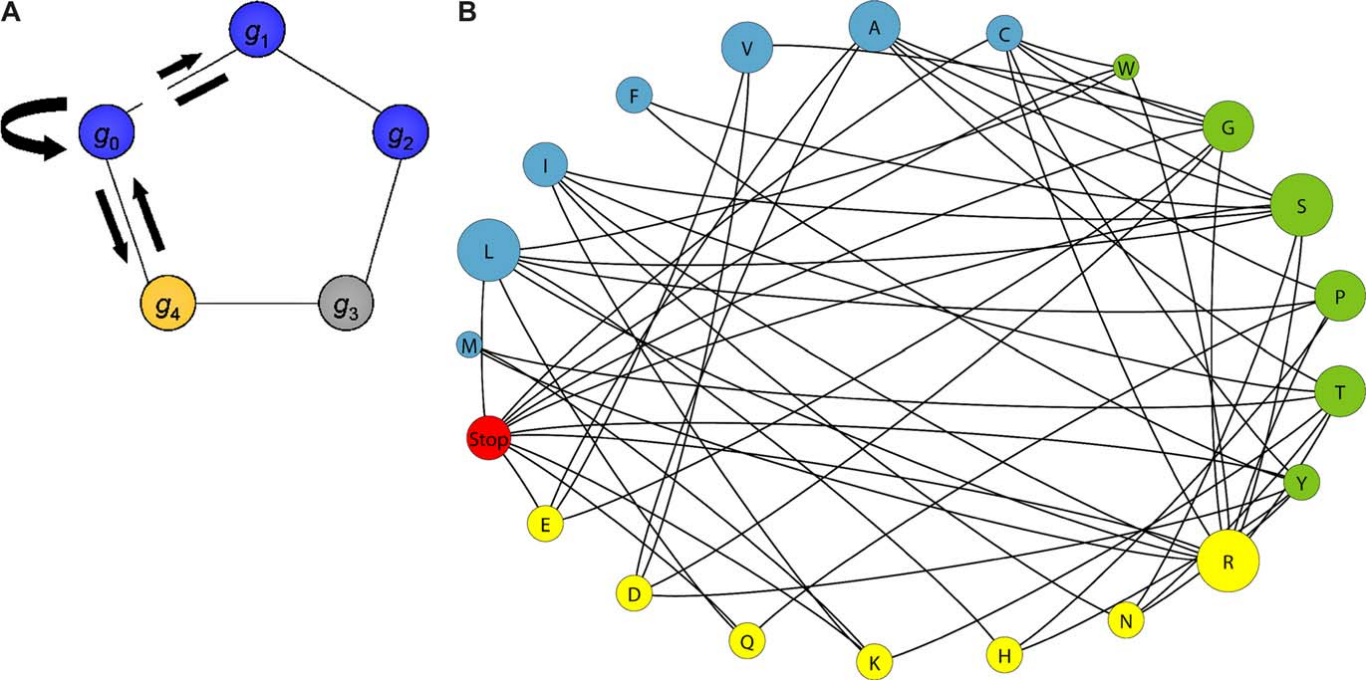
Mutational Networks (A) Five alleles lie on a mutational pentagon with genetic degeneracy for the A phenotype. Colors indicate phenotypes with blue for A, yellow for B, and gray for V. Edges indicate that an allele on one side can mutate to the allele on the other side. Arrows illustrate the dynamics in equation 2. (B) Each vertex represents an amino acid. The size of the vertex indicates the number of codons coding for the amino acid. Edges indicate point mutations between hydrophobicity classes. Mutations that preserve hydrophobicity class, including those that preserve the amino acid, are included in the model but not depicted here. The color of the vertex corresponds to the hydrophobicity class: blue indicates hydrophobic, yellow indicates hydrophilic, green indicates intermediate, and red indicates stop codons [21]. This network was drawn with PAJEK [50].

We consider the evolution of a trait in an environment that alternates between two states (E_A_ and E_B_), spending exactly λ generations per state between shifts. The simple model includes three phenotypes—one optimal phenotype for each of the two environments (A and B) and a third that has intermediate quality in both environments (V)*—*and a minimal amount of degeneracy in the relationship between the genotype and the phenotype. In particular, there is a single genetic locus, and five allelic possibilities at that locus (Figure 2A). Three of the alleles, *g*_0_, *g*_1_, and *g*_2_, give rise to phenotype A, the fourth, *g*_3_, gives rise to phenotype V, and the fifth, *g*_4_, gives rise to phenotype B. The mutational structure is a pentagon in which *g_i_* can mutate to *g*_(*i −* 1) mod 5_ or *g*_(*i* + 1) mod 5_ for *i* ∈ {0,1,2,3,4}.

The fitness function changes with the environment such that


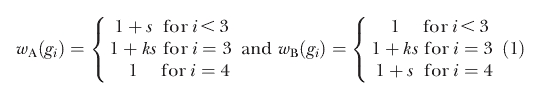


where *w*_A_ and *w*_B_ are the fitnesses in environments E_A_ and E_B_, respectively, *s* > 0 is the fitness advantage for the specialized phenotype (A or B) in its preferred environment, and 0 ≤ *k* ≤ 1 determines the intermediacy of the V phenotype.

We can write the full model as a set of difference equations


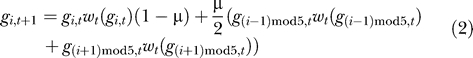


for *i* ∈ {0,1,2,3,4}, where μ is the mutation rate and *w_t_* denotes the fitness in the current environment (Figure 2A). The number of individuals with genotype *g_i_* at time *t* is denoted by *g_i,t_*. The changing environment is governed by the following rule:


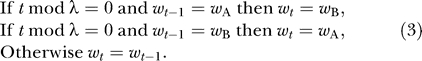


To simplify the analysis, this model tracks changes in the absolute population sizes of the various genotypes rather than their relative frequencies. Since the dynamics scale linearly with the total population size, one can achieve the same population dynamics by replacing the absolute sizes with relative frequencies and normalizing appropriately.

#### Variations on the simple model.

There are exactly 14 unique mutational networks consisting of five alleles on a pentagon, with at least one encoding A and at least one encoding B (see Materials and Methods). These include, for example, the pentagon with four consecutive alleles coding for A and one for B and the pentagon with alleles alternating in phenotype-A-B-A-V-B-. We are presenting analysis of the -A-A-A-V-B- model because it gives rise to some of the most interesting and generic dynamics found among these 14 models.

#### The codon model.

The previous model offers a transparent illustration of evolutionary dynamics under different rates of environmental change. Although somewhat simplistic, we believe that the qualitative predictions of the model will hold for a wide range of more plausible genotype–phenotype maps. To demonstrate this, we consider the evolution of a single amino acid site under fluctuating conditions. In this model, the genotypes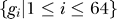 are the 64 codons in the standard genetic code and the phenotypes are hydrophobicities of the corresponding amino acids [21]. The environment alternately favors hydrophobic and hydrophilic amino acids. There are three classes of amino acids—hydrophobic, intermediate, and hydrophilic—and all amino acids in a class share the same fitness. The fitnesses are determined as in equation 1, with the fitnesses of all three stop codons equal to zero.

Each codon is mutationally connected to the nine others to which it can mutate via point mutation. This gives rise to the genetic network depicted in Figure 2B and the dynamics given by


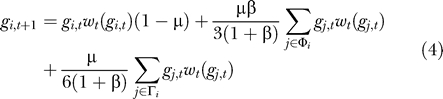


for 1 ≤ *i* ≤ 64, where μ is the overall mutation rate, β is the transition/transversion ratio (2β is the transition/transversion rate ratio), Φ*_i_* is the set of three transition point mutations of codon *i,* and Γ*_i_* is the set of six transversion point mutations of codon *i.*

### Analysis of the Simple Model

We provide an intuitive perspective on evolution in fluctuating environments using the simple model and then demonstrate the generality of the results in the codon model. The first results assume a mutation rate μ = 0.01, and fitnesses 1, 1.5, and 2 for the unfavored, intermediate, and favored phenotypes, respectively. In a constant environment, a population will equilibrate on genotypes that encode the optimal phenotype. In environment E_A_, the equilibrium relative frequencies of *g*_0_, *g*_1_, *g*_2_, *g*_3_, and *g*_4_ are 0.291, 0.412, 0.292, 0.003, and 0.002, respectively, and in environment E_B_, they are 0.005, 0.000, 0.000, 0.010, and 0.985, respectively. When there is degeneracy, as there is for phenotype A, the populations evolve genetic robustness, that is, more mutationally protected genotypes appear in higher frequency. In particular, *g*_1_, which lies in the center of the three genotypes that code for A, appears in higher frequency than either genotype on the edge of the neutral network for A (*g*_0_ and *g*_2_) at equilibrium in E_A_. In the absence of degeneracy (phenotype B), we observe a mutation–selection balance around the single optimal genotype. These findings are consistent with and provide a transparent example of the extensive theory on mutation–selection balance, quasi-species, and the evolution of genetic robustness in neutral networks [2,22–24].

**Figure 3:**
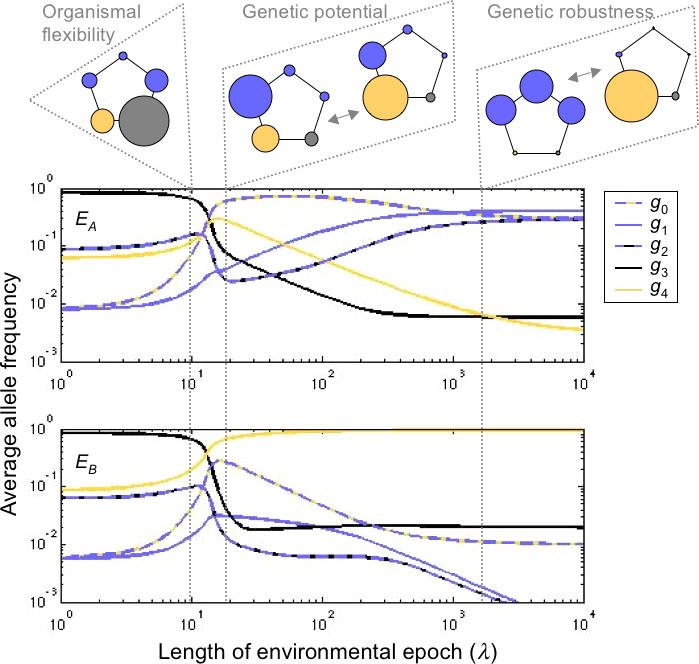
Allele Distributions under Environmental Fluctuations The graphs show the stationary allele distributions averaged over an E_A_ epoch (top) and an E_B_ epoch (bottom) as a function of the variability of the environment. As environmental variability decreases, the population moves from the intermediate phenotype to the genetic boundary between the A and B phenotypes, and eventually to an oscillation between the center of the network for A and the gene for B. Diagrams above the graphs illustrate the frequency distributions in each of the three phases. Vertex areas are proportional to the average frequencies for each allele. (For the data depicted in this figure, *s* = 1, *k* = 0.5, and μ = 0.01.)

Under rapid environmental fluctuations, populations do not have time to reach a stable allele distribution. As the environment becomes more variable, the distributions of alleles go through three distinct phases. Figure 3 shows the frequency of every allele averaged over each environmental condition after the population has reached steady oscillations. For relatively stable environments, the populations swing back and forth between near equilibrium conditions for E_A_ and E_B_, thereby alternating between genetic robustness for A and a mutation–selection balance around the single allele for B. At intermediate rates of fluctuation, populations hover near *g*_4_ and *g*_0_, where the genotypes for A abut the genotype for B. Thus, mutation between the two phenotypes occurs frequently. We call this outcome genetic potential because of the enhanced potential for mutations to give rise to novel (beneficial) phenotypes. Finally, for highly variable environments, the populations converge on the phenotype V, which has unchanging, intermediate fitness in both environments. Phenotype V corresponds to organismal flexibility—individual organisms tolerate both conditions, but neither one exceptionally well. There are a variety of mechanisms that can give rise to an intermediate phenotype including homeostasis, somatic evolution, physiological plasticity, and behavioral plasticity [7,8]. As originally predicted by Dempster [25], the ascent of V under rapid fluctuations only occurs if the fitness of V is greater than the geometric mean fitness over time for either A or B.

### Anaylsis of the Codon Model

**Figure 4:**
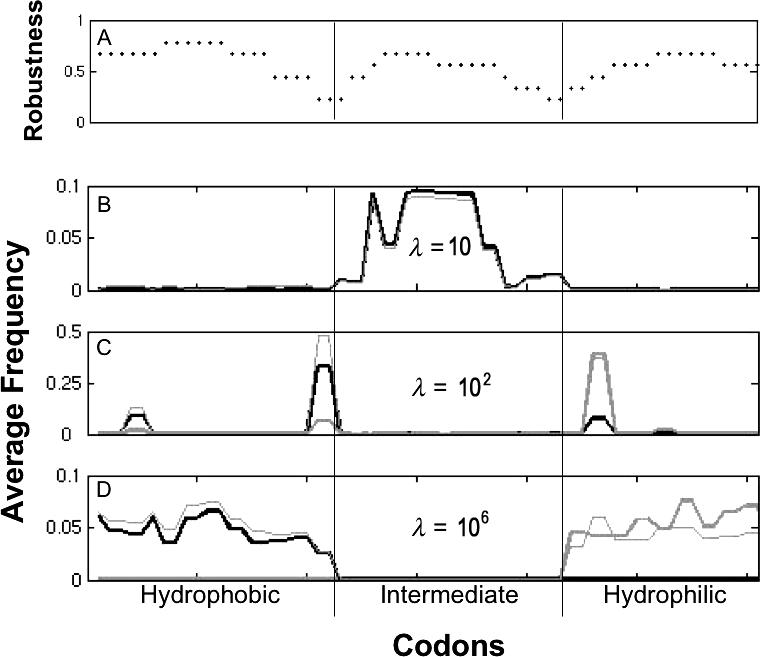
Codon Distributions under Environmental Fluctuations (A) gives the robustness for each codon, that is, the fraction of all possible point mutations that leave the hydrophobicity class unchanged. The codons have been ordered to reflect roughly the mutational adjacency of the hydrophobicity classes. (B–D) show the average codon frequency distribution for each epoch type after the population has reached stationary oscillation. These show frequencies for environmental epochs of exactly λ generations (thick lines) and epochs of random duration—Poisson distributed with mean λ (thin lines). Black corresponds to epochs favoring hydrophobicity and gray corresponds to epochs favoring hydrophilicity. The rate of environmental fluctuations is decreasing from (B) to (D) (λ = 10, 10^2^, and 10^6^, respectively).

The codon model gives rise to similar oscillations (Figure 4). Here we have assumed a transition/transversion ratio β = 2, mutation rate μ = 10^−5^, and fitnesses 1, 1.5, and 2 for the unfavored, intermediate, and favored phenotypes, respectively. (We address the impact of mutation rate in the Discussion.) Whereas in the simple model only one of the three phenotypes had multiple genotypes, in this model all three phenotypic classes have genetic degeneracy, and thus can evolve genetic robustness (Figure 4A). For highly variable environments, codons for amino acids with intermediate hydrophobicity dominate, and in particular, those that are least likely to mutate to one of the other two classes (Figure 4B). In a moderately variable environment, the populations exhibit genetic potential, hovering near the edges of the neutral networks for the two extreme classes, thereby enabling rapid evolution upon environmental transitions (Figure 4C). In relatively constant environments, we find alternating genetic robustness for the two extreme classes (Figure 4D).

**Figure 5:**
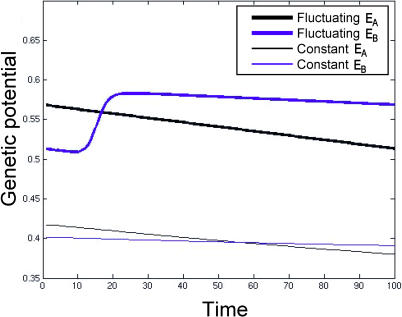
Faster Environmental Fluctuations Yield Greater Genetic Potential Genetic potential is the likelihood that a mutation to a gene coding for the currently favored phenotype will produce the intermediate or unfavored phenotype. Thick lines correspond to populations that have reached stable oscillations when λ = 100, and thin lines correspond to populations that experience a single environmental shift after having equilibrated in a constant environment. The maximum genetic potential after a single shift is significantly less than the minimum under persistent fluctuations.

The genetic potential of a population can be estimated by the probability that a currently favored codon in the population will mutate to a currently unfavored or intermediate codon. This indicates the capacity to bounce back (via mutation and selection) if and when the environment reverts. For populations that have equilibrated in a constant environment and have recently experienced an environmental shift, genetic potential will decrease as the population becomes increasingly robust to the effects of mutation (Figure 5). For populations that have evolved under moderately fluctuating conditions, genetic potential remains noticeably higher. This suggests that the regular oscillations of such populations involve distributions of codons that are quite different (more mutable) than those found during the early stages of adaptation in an isolated selective sweep.

**Figure 6:**
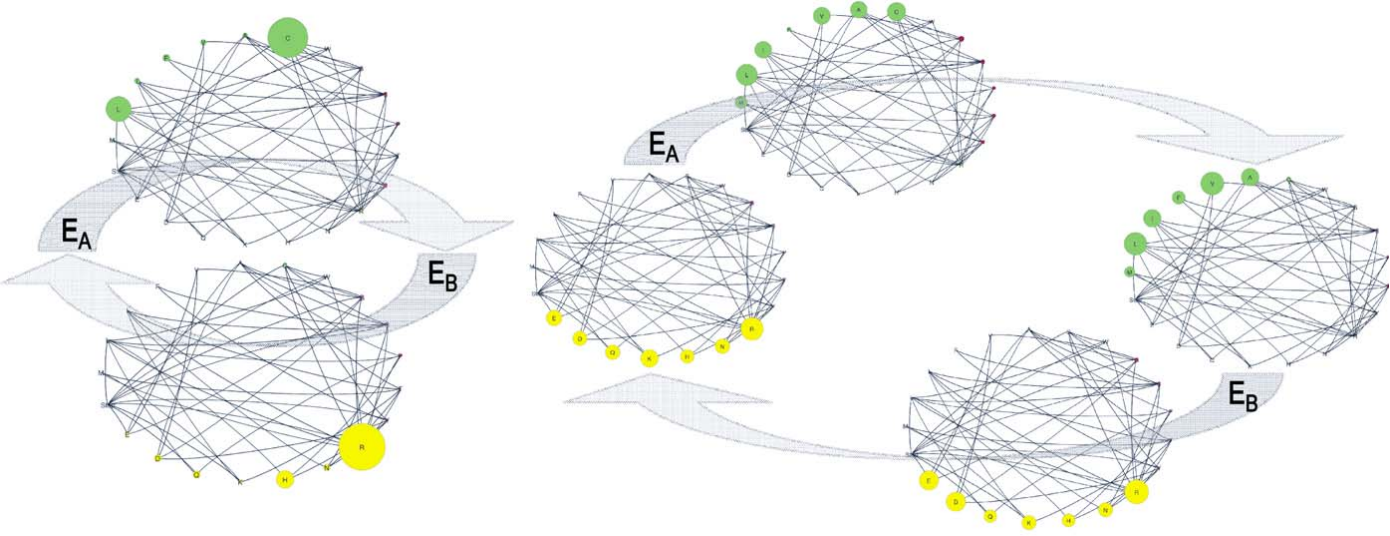
Amino Acid Distributions Reflect Genetic Potential The left figure illustrates amino acid distribution in the generations with greatest genetic potential during each of the two epochs for λ = 100. Vertex area is proportional to the relative frequency of an amino acid. The right figure gives the amino acid distributions at equilibrium in the two environments (far left and right networks), and the transitional amino acid distributions that are most similar to those depicted for λ = 100 (left). Similarity is measured as mean squared difference in frequencies across all amino acids. The amino acid networks were drawn with PAJEK [50].

This difference also appears in the distributions of amino acids. We calculated the genetic potential in each generation of a population experiencing fluctuations every λ = 10^2^ generations. Figure 6 (left) depicts the amino acid distributions for the generations that have the highest genetic potential in E_A_ and E_B_. We then compared these two distributions to the evolving amino acid distribution in a population that equilibrates in one of the two environments and then faces an environmental shift. Figure 6 (right) shows the steady state distributions for this population and the transitional distributions that are most similar (i.e., smallest average squared difference in relative frequencies) to those depicted in Figure 6 (left). The distributions of amino acids in regions of genetic potential are strikingly different than those realized in populations evolving after an isolated environmental shift.

## Discussion

We have provided an intuitive framework for studying the evolutionary implications of heterogeneous environments. Although much is known independently about the evolution of genetic robustness [3] and organismal flexibility [7,8], this model demonstrates that the extent of environmental variability may determine which of these two states evolves, and suggests the possibility of an intermediate state of heightened mutability. The transition points among the three states will be functions of both the environment and the mutation rate. In particular, increasing (decreasing) the mutation rate (within a moderate range) has the same qualitative effect as increasing (decreasing) the duration of an environmental epoch. As the mutation rate decreases, populations take longer to achieve genetic robustness, and therefore evolve genetic potential (rather than robustness) over large ranges of environmental variability. For example, at a mutation rate of μ = 10^−5^ in the codon model, populations evolve genetic potential when environment varies at rates of 10^1^ < λ < 10^6^ generations, approximately (Figure 4). If the mutation rate increases to μ = 10^−2^, the qualitative results are similar, with populations evolving genetic potential when the environmental variability is in the more limited range of 10^0^ < λ < 10^3^ generations, approximately. If, instead, the mutation rate decreases to μ = 10^−9^, then adaptation to genetic robustness proceeds at an exceedingly slow pace, yielding genetic potential throughout the extended range of 10^2^ < λ < 10^10^ generations, approximately. To understand the comparable roles of mutation and environmental variability, note that the model includes three time-dependent processes—mutation, environmental change, and population growth. If one of these rates is changed, the other two can be modified to achieve identical system behavior on a shifted time scale. Since the dynamics only weakly depend on the force of selection, we can change the mutation rate and then scale the rate of environmental change to produce the original qualitative results. The connection between environmental variability and mutation has been noted before, with theory predicting that the optimal mutation rate under fluctuating environmental conditions is μ = 1/λ [26,27].

Our results suggest an alternative perspective on the evolution of mutation rates. Theory suggests that the optimal mutation rate should correspond to the rate of environmental change [26,28], yet the extent to which mutation rate can evolve is unclear [12,13,29]. Here we suggest that the genotypic mutation rate need not evolve as long as the phenotypic or effective mutation rate evolves. By evolving toward genotypes with higher genetic potential, populations increase the rate of phenotypically consequential mutations without modifications to the underlying genetic mutational processes.

We would like to emphasize that our second model is intended as one possible example of fluctuating selection among many thought to exist in nature. Whether or not one has much confidence in the particular evolutionary scenario, the qualitatively similar outcomes for the simple and complex models presented here suggest that the results may hold for a large class of systems in which there is redundancy in the relationship between genotype and phenotype. Hydrophobicity is just one of several physicochemical properties thought to play a role in the shifting functional demands on amino acids [17–20]. Another example is phase-shifting bacteria that have mutational mechanisms, for example, inversions in promoter regions [30] and slip-stranded mispairing within microsatellites [12], that lead to variation in functionally important phenotypes. The remarkable suitability of the phase-shifting variants to the diverse conditions experienced by the bacteria suggests that phase shifting may have evolved as a mechanism for genetic potential. We hypothesize that the major histocompatibility complex (MHC), which is the component of the immune system responsible for recognizing and binding foreign particles, may also have evolved genetic potential as a by-product of the flucuations arising out of coevolution with pathogens [31]. Studies suggest that several components of the immune system exhibit high overall rates of genetic change. In particular, there are specific amino acid sites within the MHC complex that seem to have experienced rapid evolutionary change [32]. One possible explanation is that each MHC gene as a whole, and these sites in particular, have a history of rapid adaptation to changing distributions of potential antigens. We therefore predict that such sites may have evolved genetic potential.

Evolvability has been defined as a population's ability to respond to selection [6,33]. Although the term has only recently taken root, ideas concerning the evolution of evolvability itself date back to the Fisher–Wright debate over the evolution of dominance [34,35] and include the large body of theory on the evolution of mutation rates and recombination [36,37]. Developmental biologists have begun to identify genetic architectures that promote diversification [38] and buffering mechanisms, such as heat shock proteins, that allow the accumulation of cryptic variation [39]. Although one can think of genetic potential as an abstraction of all mechanisms that increase the likelihood that a mutation will have a phenotypic effect, the genetic potential that evolves in our models is a very simple form of evolvability that exploits redundancy in the map from genotype to phenotype.

Genetic potential evolves in our models because prior and future environments are identical. If, instead, the environment continually shifts to completely novel states, the evolutionary history of a population may not prepare it for future adaptation. We speculate that some degree of genetic potential may still evolve if there exist genotypes on the periphery of neutral networks with broad phenotypic lability.

Biologists often refer to phenotypic plasticity, learning, and other forms of organismal flexibility as “adaptations” for coping with environmental heterogeneity [7,8]. Should genetic potential be seen as an alternative “solution,” or should it be viewed as simply a product of fluctuating selection? Although we remain agnostic, we note that this question might be asked of all forms of adaptive variation. Whether or not genetic potential should be viewed as an evolved strategy, we emphasize that it is not simply the truncation of the adaptive path a population follows from the equilibrium state in one constant environment to the equilibrium state in the other. In the codon model, intermediate rates of environmental fluctuations push the population into regions of the codon network where genetic potential is consistently higher than the regions of network through which a population crosses after an isolated environmental shift (Figures 1, 5, and 6).

A long-standing technique for identifying selected genes is to compare the frequencies of nonsynonymous and synonymous substitutions *(K_a_*/*K_s_)* [40]. Genes experiencing frequent selective sweeps should have relatively large amounts of variation in sites that modify amino acids. Such genes might be in the process of evolving a new function or, more likely, involved in an evolutionary arms race, for example, epitopes in human pathogens [31,41] or genes involved in sperm competition [42]. In the latter case, our model suggests that, in addition to an elevated *K_a_*/*K_s_*, such genes should employ a distinct set of codons with high genetic potential. Note that this type of genetic potential is not equivalent to codon bias, but rather implies changes in the actual distribution of amino acids.

A similar argument also underlies the recent use of codon distributions for detecting genetic loci under directional selection [43]. Codon volatility—the probability that a codon will mutate to a different amino acid class, relative to that probability for all codons in the same amino acid class—is a measure of genetic potential. Genes with significantly heightened volatility will be more sensitive to mutation. Our model suggests a different explanation for codon volatility than that presented in [43]: volatility may indicate a history of fluctuating selection rather than an isolated evolutionary event. If true, then we would not expect the codon distribution to reflect a transient out-of-equilibrium distribution as the population is moving from one constant environment to another [16]. Instead, we expect the distribution to reflect the stationary level of genetic potential that corresponds to variability in the selective environment for that gene. On a practical level, therefore, the isolated selective sweep model assumed in [43] may misestimate the expected volatility at such sites. Codon volatility, however, can arise as a by-product of processes other than positive (or fluctuating) selection. It has been noted that codon volatility may instead reflect selection for translation efficiency, relaxed negative selection, strong frequency-dependent selection, an abundance of repetitive DNA, or simple amino acid biases [44–48]. Therefore, the presence of codon volatility by itself may not be a reliable indicator of either recent directional selection or fluctuating selection.

We would like to emphasize that the goal of this study was not to develop a new method for detecting positive (or fluctuating) selection, but rather to develop a theoretical framework for considering the multiple outcomes of evolution under fluctuating conditions. We conclude by suggesting an empirical method to identify loci that have evolved genetic potential under such conditions as distinct from those that have experienced a recent selective sweep. Suppose that a gene experiences fluctuations at a characteristic rate across many species. Furthermore, suppose that multiple sites within the gene are influenced by such fluctuations. For example, there may be fluctuating selection for molecular hydropathy, charge, size, or polarity, and several sites within the gene may contribute to these properties. Such sites should evolve in tandem and equilibrate on similar levels of genetic potential, and thus exhibit similar codon (and amino acid) distributions across species. In contrast, if a gene experiences isolated selective sweeps, then the variation at all sites should correspond to both the history of selective events and the species phylogeny, and the amino acid distributions at sites should correlate only when sites functionally mirror each other. Thus, one can seek evidence for the evolution of genetic potential as follows. First, identify genes that are rapidly evolving, perhaps by calculating *K_a_*/*K_s_* ratios. Such sites have been identified, for example, in human class I MHC genes, the HIV envelop gene, and a gene from a human T cell lymphotropic virus (HTLV-1) [31,32]. Within these genes, search for sites for which there is minimal correlation between the species tree and the amino acid distribution. Our model predicts that some of these sites should share similar distributions of amino acids across species.

## Materials and Methods

### Mathematical analysis of models.

For the two models, we calculate the deterministic, infinite population allele frequency distributions in constant and fluctuating environments. Let **M**_A_ and **M**_B_ be the normalized transition matrices that govern changes in the allele frequencies in E_A_ and E_B_ epochs, respectively. The entries in these matrices are defined by equations 2 and 4. The left leading eigenvectors for **M**_A_ and **M**_B_ give the equilibrium frequency distributions of alleles in each of the two constant environments, respectively. Under fluctuating conditions with epoch duration of λ generations, we iteratively apply the matrices, and then compute the left leading eigenvector of 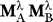 . This vector, which we call ***v***_B_, gives the allele frequency distribution at the end of an E_A_ epoch followed by an E_B_ epoch.

We are interested not only in the final allele distributions, but also in the dynamics throughout each epoch. Thus, we calculate the average frequency of each allele across a single E_A_ epoch by


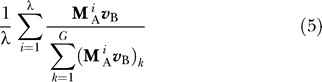


where *G* is the total number of alleles in the model (*G* = 5 for the simple model and *G* = 64 for the codon model) and the subscript *k* indicates the *k*th entry in the vector. Similarly, the average distribution across an E_B_ epoch is given by


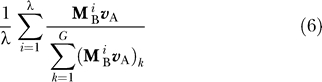


where ***v***_A_ is the allele frequency distribution at the end of an E_B_ epoch followed by an E_A_ epoch and is equal to the left leading eigenvalue of 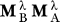


For the codon model, we compare these calculations that assume a regularly fluctuating environment to numerical simulations that assume a Poisson distribution of epoch lengths. In each generation of the simulations, the environmental state switches with probability 1/λ and the codon frequencies are then multiplied by the appropriate transition matrix.

### Proof of 14 unique pentagonal networks.

**Figure 7:**
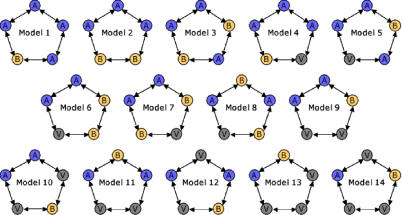
Pentagonal Mutational Networks These are the 14 possible pentagonal mutational networks consisting of five alleles producing phenotypes A, B, or V, with at least one encoding A and one encoding B.

We use an elementary group theoretic result known as Burnside's Lemma [49] to prove that there are 14 distinct mutational networks consisting of five alleles on a pentagon that map to the set of phenotypes {A, B, V} and contain at least one of each specialist phenotype (A and B) (Figure 7). We assume that all rotations and reflections of a network are equivalent to the original network, and that A and B are interchangeable. For example, the six networks with phenotypes -A-A-A-B-B-, -B-A-A-A-B-, -B-B-A-A-A-, -B-B-B-A-A-, -A-B-B-B-A-, and -A-A-B-B-B- are equivalent.

Let *X* be the set of all pentagons with vertices labeled {A, B, V} having at least one A vertex and at least one B vertex. The size of *X* is the number of all pentagons with labels {A, B, V} minus the number of pentagons with labels {A, V} or {B, V}, that is, |*X*| = 3^5^ − (2 · 2^5^ − 1) = 180.

We define the group *G* of all actions on *X* that produce equivalent pentagons (as specified above). *G* is made up of (1) the identity, (2) the four rotations and five reflections of the pentagon, (3) interchanging all As and Bs, and (4) all the combinations of the above actions. Thus *G* is equal to the 20-member group {ι, ρ, ρ^2^, ρ^3^, ρ^4^, σ_0_, σ_1_, σ_2_, σ_3_, σ_4_, α, αρ, αρ^2^, αρ^3^, αρ^4^, ασ_0_, ασ_1_, ασ_2_, ασ_3_, ασ_4_} where ι is the identity, ρ is a single (72°) rotation, σ*_i_* is a reflection through vertex *i,* and α is replacement of all As with Bs and all Bs with As. (Note that the reflections are rotations of each other, for example, ρ^2^σ_0_ = σ_1_.)

The number of distinct mutational networks is equal to the number of orbits of *G* on *X*. Burnside's Lemma tells us that this number is


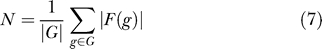


where *F*(*g*) = {*x* ∈ *X* | *gx* = *x*} is the set of fixed points of *g*. For each of the twenty elements of *G*, we exhaustively count *F*(*g*).

The identity fixes all elements of *X,* that is, *F*(ι) = *X*. Each of the various rotations of a pentagon (through 72°, 144°, 216°, and 288°) has the property that its iterations move a given vertex to every other vertex of the pentagon without changing the letter assigned to that vertex. The same is true of the square of the product of any rotation and an A–B flip. Hence, any fixed point of one of these elements of the group *G* would necessarily have the same label at each vertex of the pentagon. Since every labeled pentagon in *X* has at least one A label and at least one B label, then no element of *X* has the same label at each vertex. Thus, the fixed point set of every rotation and of every product of a rotation and an A–B flip must be empty, that is, *F*(ρ*^n^*) = *F*(αρ*^n^*) = ∅︀ for all *n*. By a similar argument, the simple A–B flip also has no fixed points. Every reflection fixes 12 elements of *X,* for example,


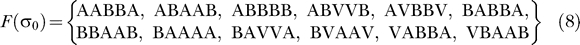


and every product of a reflection and an A–B flip fixes eight elements of *X,* for example,


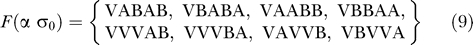


In sum, all eight group elements that involve rotations fix no elements of *X,* all five reflections fix 12 elements of *X,* and all five combinations of a reflection and an A–B exchange fix eight elements of *X*. Thus,


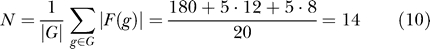


## Abbreviation

MHC: major histocompatibility
